# Detecting unexpected growths in health technologies expenditures: the case of MIPRES in Colombia

**DOI:** 10.1186/s12913-023-10155-w

**Published:** 2023-10-25

**Authors:** Oscar Espinosa, Valeria Bejarano, Cristian Sanabria, Jhonathan Rodríguez, Sergio Basto, Paul Rodríguez-Lesmes, Adriana Robayo

**Affiliations:** 1 Directorate of Analytical, Economic and Actuarial Studies in Health, Instituto de Evaluación Tecnológica en Salud (IETS), Bogotá, D.C Colombia; 2 Economic Models and Quantitative Methods Research Group, Centro de Investigaciones para el Desarrollo, Universidad Nacional de Colombia, Bogotá, D.C Colombia; 3 School of Economics, Universidad del Rosario, Bogotá, D.C Colombia; 4 Executive Directorate, Instituto de Evaluación Tecnológica en Salud (IETS), Bogotá, D.C Colombia

**Keywords:** Health technologies, Health expenditures, High-cost technology, Enteral nutritional, Statistical data analysis, Data analytics

## Abstract

We developed an algorithm to explore unexpected growth in the usage and costs of health technologies. We exploit data from the expenditures on technologies funded by the Colombian government under the compulsory insurance system, where all prescriptions for technologies not included in an explicit list must be registered in a centralized information system, covering the period from 2017 to 2022. The algorithm consists of two steps: an outlier detection method based on the density of the expenditures for selecting a first set of technologies to consider (39 technologies out of 106,957), and two anomaly detection models for time series to determine which insurance companies, health providers, and regions have the most notorious increases. We have found that most medicines associated with atypical behavior and significant monetary growth could be linked to the use of recently introduced drugs in the market. These drugs have valid patents and very specific clinical indications, often involving high-cost pharmacological treatments. The most relevant case is the Burosumab, approved in 2018 to treat a rare genetic disorder affecting skeletal growth. Secondly, there is clear evidence of anomalous increasing trend evolutions in the identified enteral nutritional support supplements or Food for Special Medical Purposes. The health system did not purchase these products before July 2021, but in 2022 they represented more than 500,000 USD per month.

**Supplementary Information:**

The online version contains supplementary material available at 10.1186/s12913-023-10155-w.

## Introduction

The rapid growth of health expenditures is one of the central concerns of health systems [1]. While it is well known that adopting new technologies is the primary source of such growth [2], unexpected sources can undermine the goal of providing healthcare cost-effectively. In this article, we propose a methodology to detect abnormal sources of growth based on outlier detection techniques. To achieve this, we use administrative records collected for monitoring health technology purchases at the national level.

One of the main goals of health information systems is to improve the efficiency of healthcare. While most of the literature has focused on improving preventive health interventions by collecting clinical data, an alternative but less explored objective is to leverage information on technology usage and costs to achieve economic goals [3–7]. In this context, the routine incorporation of statistical techniques that detect relationships in historical data – commonly known as data mining – emerges as a promising strategy to have information readily available for decision-making [8].

Currently, most applications revolve around predicting the future costs associated with treating well-known conditions using standard technologies [9, 10]. Nevertheless, there is a lack of studies on the costs of new technologies or new uses of existing ones. Colombia, a country with a comprehensive health benefits package (HBP) and universal healthcare, operates under a managed competition compulsory insurance system. Insurance companies purchase healthcare technologies and provide services using two sources of capitation-based resources transferred by the government. Most technologies and resources are managed under an explicit HBP list, which undergoes periodic updates. In addition, there exists another list of technologies and services not included in the HBP list.

Prescribers can request these technologies for individual cases using an information system known as MIPRES [11].^1^ The MIPRES system centralizes all invoices for each specific health technology, regardless of the insurance company or the health provider involved in the transaction.^2^ We leverage this information to detect unexpected sources of growth in individual health technologies. This algorithm provides the Colombian government with a tool to oversee the utilization of healthcare resources for technologies that have not yet undergone regular inclusion into the HBP explicit list.

## Methods

### Data

We utilize information from MIPRES, provided by the Ministry of Health and Social Protection (MHSP), which includes data on all technologies outside the HBP explicit list that are eventually provided to patients. These technologies represent nearly 5% of the country’s healthcare budget. The dataset includes standardized technology code,^3^ technology type (medication, health procedure, medical devices, enteral nutritional support products, and complementary services), unique patient anonymized identifier, place of residence,^4^ the insurance company identification, the health provider identification, the date, and the International Classification of Diseases 10th Revision (ICD-10) code of the health condition for which the technology is requested.

We aggregated the data quarterly, covering the period from January 2017 to March 2022, resulting in a dataset of 106,957 technologies. For our analysis, we focus on three variables: total costs, total unique users, and cost per capita. The statistical applications of this research are developed in R software, version 4.2.3.

Our data shows a significant increase in the number of people who have received technologies not listed in the HBP between 2017^5^ and 2021 (178% increase, from 826,298 people in 2017 to 2,297,314 in 2021). Additionally, there has been a 24.5% increase in the diversity of health conditions attended, rising from 6,368 health conditions in 2017 to 7,739 in 2021.

In monetary terms, there was an increase in spending per individual; however, this trend declined in 2020, likely due to the pandemic’s effect. This suggests that, although more people were attended to, the technologies provided were of lower value compared to other years. Notably, spending per health condition (annual amount per ICD-10 code) has seen a significant growth of just over 322% between 2017 and 2021. Table 1 presents overall information disaggregated by technology type.

**Table 1:** Characterization of the dataset

Year	Variable	Medications	Health procedures	Medical Devices	Nutritional support products	Complementary services
**2017**	Frequency of technologies	2,573,912	108,402	12,259	81,154	107,510
	Unique diseases	8,501	2,869	499	2,112	2,324
	Unique users	745,432	72,169	2,788	38,641	34,767
	Cost per capita	$486.2	$587.1	$231.6	$475.9	$387.0
**2018**	Frequency of technologies	9,853,985	413,152	2,182	321,481	525,952
	Unique diseases	6,761	3,990	189	3.090	3,576
	Unique users	1,490,462	224,066	685	92,272	84,055
	Cost per capita	$770.3	$457.3	$171.5	$769.8	$666.6
**2019**	Frequency of technologies	12,969,785	388,107	116	515,204	1,161,415
	Unique diseases	6,875	3,874	56	3,420	4,399
	Unique users	1,796,901	152,439	95	143,773	145,019
	Cost per capita	$801.1	$668.9	$404.3	$640.0	$999.8
**2020**	Frequency of technologies	13,161,867	1,291,945	117	647,580	1,545,325
	Unique diseases	6,950	4,193	43	3,683	4,623
	Unique users	1,869,172	1,003,328^a^	83	198,196	201,034
	Cost per capita	$862.1	$164.2	$169.9	$552.3	$969.6
**2021**	Frequency of technologies	12,550,695	261,139	195	850,461	1,964,561
	Unique diseases	6,891	4,001	60	3.836	4,830
	Unique users	1,967,276	168,072	175	267,451	253,225
	Cost per capita	$907.0	$642.1	$173.2	$484.8	$947.7
**2022 (only Q1)**	Frequency of technologies	356,776	3,756	21	158,973	339,637
	Unique diseases	3,057	652	14	2,258	3,091
	Unique users	206,315	3,389	16	93,554	150,710
	Cost per capita	$455.7	$396.2	$155.1	$241.9	$208.4

Costs in USD (exchange rate of 3,500 COP per 1 USD)

Source: own elaboration based on MIPRES information provided by the Ministry of Health and Social Protection

^a^The increase that is registered for this year is related to Resolution 894 of 2020 which indicates "The health service provider institutions, through the personnel they authorize, may report in the MIPRES technological tool, the rapid search and screening tests, as well as the molecular diagnostics for SARS-CoV-2 prescribed in the outpatient setting since April 2, 2020 and during the period of the health emergency due to the coronavirus COVID-19″ [12]

### Empirical strategy

**Fig. 1:**
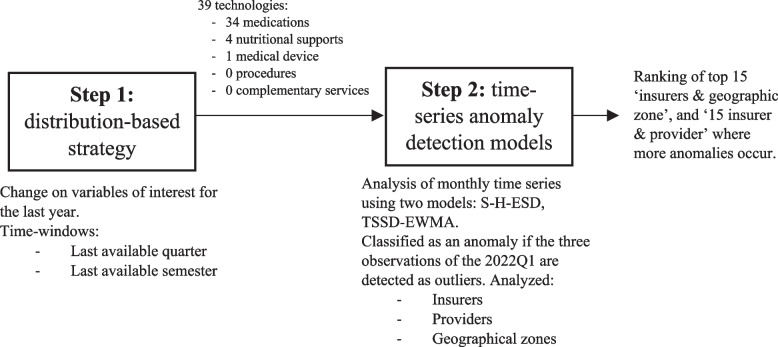
Diagram of the algorithm for the selection of health technologies and results

We considered several alternatives of anomaly detection methods in the statistical literature (see Appendix 2 for a short review). Based on our review, we have defined two steps for detecting anomalies in the growth of healthcare usage for the first quarter of 2022 (2022Q1) for each type of health technology (see Fig. 1):

1. Following Tiwari et al. [13] flooring and capping method, we selected technologies in the 99th percentile of highest change on the variables of interest. To define change over time, considering differences in frequencies of administration and the value of each technology, we utilized two time windows^6^:
  i. Quarter: January to March 2022 (2022Q1) compared to January to March 2021 (2021Q1).
  ii. Semester: October 2021 to March 2022 (2021Q4-2022Q1) compared with October 2020 to March 2021 (2020Q4-2021Q1).

Next, we selected technologies that appeared in all six possible combinations (3 variables $x$ 2 time windows) based on two characteristics: prioritization and parsimony.

2. We applied two time series anomaly detection models to the monthly time series of the selected technologies, disaggregated at ‘insurer & geographic zone’, and ‘insurer & provider’. The models used were Seasonal Hybrid Extreme Studentized Deviate (S-H-ESD) and Two-Stage Dataset Shift-detection based on an Exponentially Weighted Moving Average (TSSD-EWMA). These techniques are commonly employed and known for their robustness in handling non-stationary time series [14–19]. The final set of anomalies consists of the series in which all three observations in 2022Q1 are detected as outliers by both algorithms.

## Results

### Anomalies detection

Figure 1 provides an overview of the analysis process and the corresponding findings. From step 1, we identified 39 technologies exhibiting anomalies in their change over time. These selected cases encompassed 34 medications, four nutrition products (nutritional support supplements in drinks or powder), and one medical device. Notably, no anomalies were detected among procedures and complementary services.

It has been established that the majority of the analyzed medicines, which have received approval from the local Food and Medicines Administration Agency (INVIMA), are indicated for specific clinical uses with recent issue dates on their registrations. These medications belong to pharmacological groups used to treat various conditions, including rare diseases, autoimmune and genetic conditions, cancer, chronic diseases, obesity, and epilepsy, among others. Most of these products are associated with a single producer or importer, and some are part of a special INVIMA list. This list includes vital medicines that are unavailable for specific conditions but are known to be in short supply [20]. Notably, five of these medicines are classified by the European Medicines Agency (EMA) as orphan medicines, designated for the treatment of rare conditions that affect five out of every 10,000 individuals.

Regarding the identified enteral nutrition products (nutritional support supplements), INVIMA classifies them as Foods for Special Medical Purposes (FSMPs) in Colombia. The FSMPs are products designed and manufactured to provide total or partial nutritional support to patients with diseases or medical conditions that require special nutritional requirements beyond what can be achieved by modifying a conventional diet alone. These products can be administered orally or through a tube at various levels of care, including hospitals, outpatient settings, or even at home [21, 22].

Lastly, the medical device corresponds to the purchase of external glass or plastic corrective lenses to address reduced visual acuity. It is essential to note that these lenses are part of the HBP explicit list, allowed once a year for individuals up to 12 years of age, and only once every five years for those over 12 years of age [23]. This finding suggests the possibility that patients older than 12 may be requesting more frequent lens changes within a five-year period.

As a second step, for these 39 technologies, we constructed monthly time series at the ‘insurer & geographic zone’ and ‘insurer & provider levels’. Subsequently, we ranked the top 15 pairs of each type with the most detected anomalies. Three insurance companies were consistently present in the rankings, with department capitals being more common than other types of geographic areas. Surprisingly, no particular health provider stood out as responsible for the majority of atypical changes.

Regarding cost, the highest values came from 27 prescriptions of one medication (Burosumab 20 mg) with a total value of USD 307,885, a product approved in 2018 to treat a rare genetic disorder affecting skeletal growth.

### Case study: enteral nutritional support supplements

**Fig. 2:**
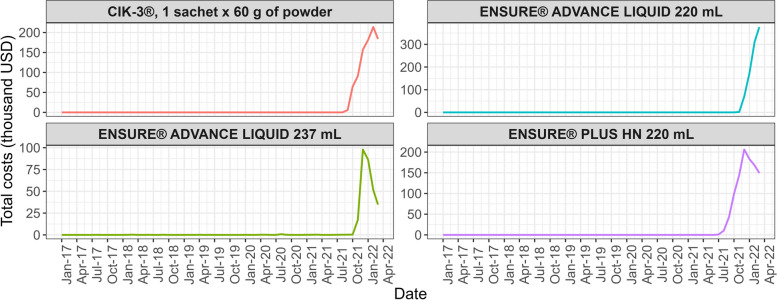
Time series for enteral nutritional support supplements Note: costs are in USD (exchange rate of 3,500 COP per 1 USD)

**Fig. 3:**
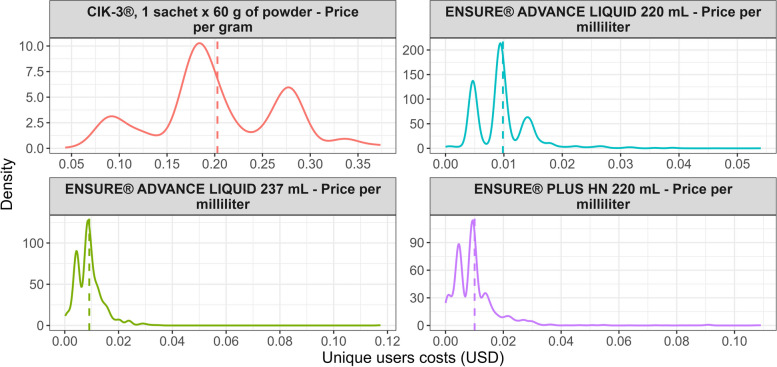
Unique users’ costs of enteral nutritional support supplements (We assume, for simplicity and based on nutritionist advice, that each record refers to 60 sachets of CIK-3® or 60 Ensure® bottles in their three variations per month since this information is not recorded in the database. However, it's important to note that the consumption of FSMPs depends on the clinical condition of the patient and the decision of the physician.) Note: the dashed vertical line is the mean cost value

As described above, four FSMPs or enteral nutritional support supplements exhibited abnormal growth, and Fig. 2 presents their respective time series. It is noteworthy that these products were not purchased with this source of resources before July 2021, but in 2022, they cost the health system more than 500,000 USD per month. Additionally, Fig. 3 illustrates a wide dispersion in the price paid per milliliter (mL) of Ensure® Advance in its various versions.

Firstly, Ensure® Advance Liquid^7^ (in its two presentations, 237 mL and 220 mL) is a hyperprotein polymeric formula and Ensure® Plus HN^8^ (220 mL) is a hypercaloric polymeric formula. These FSMPs are used to restore or maintain body mass in people with moderate or severe protein and calorie malnutrition. These formulas are mainly associated with diseases such as acquired immunodeficiency syndrome (AIDS), neurological diseases, cancer, and patients who have undergone trauma or major surgery [26].

Secondly, the powdered polymeric formula CIK-3®^9^ (1 sachet containing 60 g of powder and 1 package containing 15 sachets, equivalent to 900 g of powder) is particularly utilized in the nutritional treatment of adults to expedite the healing of chronic wounds, such as ulcers or surgical wounds, in other words, this formula was designed for consumption by adults with difficult to heal wounds [28]. It should be noted that these formulas are prescribed by the treating physician or specialist only when nutritional needs cannot be met with a normal or modified diet [29].

## Discussion

This paper explores the significant price growth of certain health technologies not included in the HBP explicit list in recent quarters, as recorded through the MIPRES tool. Increases that could be considered atypical were characterized to provide analytical tools for policymakers. Based on a 2-stage methodology, we detected 39 technologies with significant and anomalous monetary growth. These technologies were characterized at the level of insurer-geographical area and insurer-provider. Most of the cases are related to treatments for orphan medicines and other medicines for specific conditions known to be in short supply. Hence, abnormal growth in such cases can be attributed to the international market.

Considering the applications of the analyzed polymeric formulas or FSMPs, it is evident that these products are increasingly being utilized as a complementary treatment for various chronic diseases and health conditions. In this regard, it is worth contemplating their inclusion in the HBP explicit list (or their exclusion, if technical analyses determine that they do not provide value for money). Additionally, exploring centralized purchasing strategies might lead to better prices in the market without favoring specific brands or product presentations [30].

The main limitation of this research article is the restriction in presenting detailed information on the findings of the proposed methodology (names of HPE, cities, and health service providers, among others). This is due to the confidentiality of information requested by the contracting entity of the study. Furthermore, while our methodology aims to identify anomalous growths, it would be ideal to understand the underlying causes. However, it is well known that pricing by the pharmaceutical industry -or other technology providers- includes many components that are not commonly known, making it impossible to discern the exact reasons for the behavior of their cost function. Despite these limitations, this scientific article presents the first analytical approach to examining the evolution of expenditure on health technologies not included in the explicit HBP in Colombia.

## Conclusions

The rapid growth in the availability of electronic health records (EHR) presents an opportunity for health systems to improve their efficiency. However, the adoption of EHR systems does not always lead to cost reductions [7], and in some cases, the impact varies among different organizations within the same intervention [31]. Our results indicate that implementing the regular usage of statistical techniques, in conjuction with data collection, can improve efficiency by enabling the early detection of unexpected patterns in health technology consumption.

Three key elements can be concluded from the results of this analytical exercise. First, the majority of medicines with detected atypical behavior and significant monetary growth can be attributed to recently introduced medicines in the market, which hold valid patents and have highly specific clinical indications involving high-cost pharmacological treatments. Secondly, it becomes evident that the FSMPs identified are experiencing anomalous increasing trend evolutions. Third, the discovery of outliers in the ex-post review of MIPRES data underscores the necessity of establishing a predictive mechanism to raise red flags and proactively devise strategies to prevent potential excessive spending. Furthermore, integrating other sources of information into the analysis would be ideal to uncover potential reasons for unexpected increases.

Potential alternatives to control spending without compromising the quality of patient care will depend on the underlying reasons behind the increases. Alternatives, such as inclusion in the price cap mechanism based on international price benchmarking and centralized purchasing (through auctions or direct negotiation, depending on the presence or absence of generics in the market), can be promptly considered in response to specific scenarios arising from supply-side phenomena [32–34]. These scenarios encompass price hikes due to reduced competition stemming from the exit of a key supplier from the market, shortages of certain products due to external logistical or input-related shocks, or the successful marketing of new technologies across the country (e.g. specific brands of nutritional supplements), among others.

Understanding not only the atypical growth but also its potential sources will enable faster and more appropriate policy responses. The key lies in harnessing information systems through real-time analytics, integrating diverse sources of information, and tapping into the expertise of the country’s leading institutions. With this statistically robust tool based on real-world evidence, we hope that the Colombian government will be able to make resource optimization decisions in favor of maximizing the health of all Colombia’s inhabitants.

### Supplementary Information

**Additional file 1: Appendix 1.** Development of MIPRES. **Appendix 2.** Literature review on anomaly detection methods.

## Data Availability

The anonymized datasets generated and/or analyzed during the current study are not publicly available due as it is part of a confidential database of the Ministry of Health and Social Protection of Colombia, but can be made available from Corresponding Author on reasonable request.

## Footnotes

1 Aside from the HBP explicit inclusion list, the country also operates an explicit exclusion list. All other technologies could potentially be requested through MIPRES by any physician, dentist, optometrist, or nutritionist who is providing healthcare through the health system. See Appendix 1 for further details on the development of MIPRES and the purchase of technologies outside the HBP explicit list.

2 For a detailed explanation of the MIPRES system, its history, functioning, among other features, see https://www.sispro.gov.co/central-prestadores-de-servicios/Pages/MIPRES.aspx.

3 In Colombia, health services have a unique code known as *Clasificación Única de Procedimientos en Salud* (CUP), and medications have unique codes per product known as *Código Único de Medicamento* (CUM). The CUM identifies individual commercial products, so there could be several CUM with the same Anatomical Therapeutic Chemical Code – Chemical substance level (ATC5) which identifies the active principle of a medication.

4 Sixty-three geographical zones based on *municipios*, the basic administrative division of the country (similar to a county in the US): the capital of each department (equivalent to a state in the US) – 32 –, the rest of the municipalities that made up each department of the country – 30, given that Guainía and Vaupés only presented data in their respective capitals –, and a 'No identification' category – 1 –.

5 In 2017, only the Health-Promoting Entities (HPE) -i.e. health insurers in Colombia- record of the contributory regime is available. As of 2018, HPE information is recorded for both the contributory and subsidized regimes (currently, in 2023, more than 24 million people are affiliated with each health regime).

6 We do not consider growth rate as for some technologies frequencies in the first period could be close to zero, resulting in large percentage increases for relatively uncommonly used technologies.

7 Ensure® advance liquid contain HMB (Beta-Hydroxy-Beta-Methylbutyrate), protein, carbohydrates, lipids, vitamins, and minerals. Free of lactose and gluten [24].

8 Ensure® plus HN contain water, maltodextrin, caseinates, vegetable oil, soy protein isolate, minerals, stabilizers, emulsifier, and vitamins. This formula contains milk and soy ingredients [25].

9 CIK-3® contain protein way isolate, with arginine, glycine, vitamins, and minerals [27].
