# Supplementary material for: Detecting unexpected growths in health technologies expenditures: the case of MIPRES in Colombia
Source: BMC Health Serv Res. 2023 Oct 25;23:1153. doi: 10.1186/s12913-023-10155-w (PMC10601102; doi:10.1186/s12913-023-10155-w)
Supplement: Supplementary file 1 — Additional file 1: Appendix 1. Development of MIPRES. Appendix 2. Literature review on anomaly detection methods. [file 12913_2023_10155_MOESM1_ESM.docx]

# Appendix 1. Development of MIPRES

The explicit list is covered under a yearly capitation-transfer system known as Capitation Payment Unit (UPC, for its acronym in Spanish), which fund the original explicit HBP of the country. The list matches technologies with health conditions, so technologies must be approved for all potential uses. Technologies in the list were automatically approved to be used in treatment, but non-included technologies could be used under a system known as *recobros*: non-included technologies (and/or uses) that health providers considered essential for individual treatments were purchased directly by health providers. As such technologies were outside the provider’s contract with the insurance companies, they had to be approved first by a medical committee in the insurance company. Then, insurance companies requested such resources from the government via a reimbursement form (a *recobro*, in Spanish) to pay the health providers.

The MIPRES technology system was introduced in 2016 to speed up such complex requests involving several health system actors. The *recobros* system generated severe problems in terms of managing resources across the health system. Moreover, the system removed the incentives for insurance companies to contain the costs of those technologies (1).

In response, in 2019, the Colombian government generated a parallel capitation transfer system (*Presupuestos Máximos*) where insurers received ex-ante transfers to purchase those technologies outside the UPC list (2). However, the usage of the MIPRES system remained compulsory. In addition, spending on health technologies (which are reported in MIPRES) remains unchecked and is putting increasing pressure on the government's health budget.

# Appendix 2. Literature review on anomaly detection methods

Recent years have seen a significant increase in the amount of data available for analysis. This can be explained mainly by technological progress and the need of the private sector, governments, and other organizations to collect information on various indicators. The data collected come in different forms, mainly cross-sectional structures, panels, and time series, and it is the latter that is of particular interest in this study.

There are multiple approaches to time series analysis in the literature; however, the detection of outliers in time series has become a topic of interest to researchers due to the implications in fields such as econometrics, finance, signal processing, health care, industrial processes and, recently, internet traffic (3). The main focus within the statistical discipline has been on determining whether an apparently anomalous value can be classified as not being generated by the underlying statistical distribution, resulting in studies on rules for rejecting spurious observations as was done early on by Anscombe et al. (4) in the 1960s.

Papers related to time series anomaly detection can be divided into two categories: those focused on univariate time series and those dealing with multivariate time series. On the one hand, some use the histogram method and, utilizing an algorithm, detect those points -anomalies- in the series whose elimination results in an improvement in the histogram representation error of the data concerning its original form done by Jagadish, Koudas & Muthukrishnan (5) and Muthukrishnan, Shah & Vitter (6). On the other hand, there are density-based methods, which use as an anomaly classification criterion the data points with several neighbors less than a defined threshold, using some distance metric (e.g. Euclidean distance) and, to deal with the temporality of the data, the series is segmented into moving windows (7,8). However, given the structure of the time series, the methods discussed so far do not tend to dominate the literature on univariate series, with inferential model-based techniques being the predominant ones for anomaly analysis and detection (9).

The techniques based on inferential models are divided into those related to estimation and prediction, the fundamental difference between both lies in the fact that the algorithms based on estimation use the entire time horizon of the series to adjust the model, forecast the intra values and perform the anomaly detection analysis -where approximations such as the probabilistic exponentially weighted moving average (P-EWMA) (10), those based on the Box-Jenkins methodology (11,12), among others, are found-. On the other hand, prediction-based algorithms adjust the model in such a way that the predicted value is not part of the set of adjustment observations so that in practice, the latter can be applied to time series that are fed with real-time observations, thus evaluating the new information. Therefore, they have applications commonly related to streaming data, from the evaluation of environmental and spatially distributed data (13,14) to web-generated data (15).

Although univariate analysis is more common, researchers are sometimes confronted with multivariate time series data, and given that in such a scenario some particularities are encountered - the presence of some degree of correlation between variables may be one - there is a range of methods in the literature other than those collected so far in this paper. As in the univariate case, model-based techniques are more developed. Regarding prediction algorithms, there is the work of Zhou et al. (16) who use Markov processes to study multivariate time series with temporal correlation to detect anomalies or impute missing observations. On the other hand, Sakurada et al. (17), with an algorithmic estimation approach, use machine learning tools to analyze anomalies in correlated time series. Finally, there are the works of Cheng et al. (18) and Cheng et al. (19) who detect anomalies in noisy time series using Kernel and graph alignment methods.

**References**

1. Torregroza Z. Recobros en el sistema de salud de Colombia. Universidad Santo Tómas; 2018.

2. Ministerio de Salud y la Protección Social de Colombia. Así va el acuerdo de punto final. 2020.

3. Hochenbaum J, Vallis O, Kejariwal A. Automatic anomaly detection in the cloud via statistical learning. 2017; Available from: http://arxiv.org/abs/1704.07706

4. Anscombe F, Guttman I. Rejection of outliers. Technometrics. 1960;2(2):123.

5. Jagadish H, Koudas N, Muthukrishnan S. Mining deviants in a time series database. In: Proceedings of the 25th international conference on very large data bases. 1999. p. 102–13.

6. Muthukrishnan S, Shah R, Vitter J. Mining deviants in time series data streams. In: Proceedings 16th International Conference on Scientific and Statistical Database Management, 2004. 2004. p. 41–50.

7. Angiulli F, Fassetti F. Detecting distance-based outliers in streams of data. Int Conf Inf Knowl Manag Proc. 2007;811–20.

8. Angiulli F, Fassetti F. Distance-based outlier queries in data streams: the novel task and algorithms. Data Min Knowl Discov. 2010;20(2):290–324.

9. Blázquez-García A, Conde A, Mori U, Lozano J. A review on outlier/anomaly detection in time series data. ACM Comput Surv. 2021;54(3).

10. Carter K, Streilein W. Probabilistic reasoning for streaming anomaly detection. In: 2012 IEEE Statistical Signal Processing Workshop (SSP). 2012. p. 377–80.

11. Akouemo H, Povinelli R. Time series outlier detection and imputation. 2014 IEEE PES Gen Meet | Conf Expo. 2014;

12. Mehrang S, Helander E, Pavel M, Chieh A, Korhonen I. Outlier detection in weight time series of connected scales. Proc - 2015 IEEE Int Conf Bioinforma Biomed BIBM 2015. 2015;1489–96.

13. Zhang Y, Hamm, Meratnia N, Stein A, van de Voort M, Havinga P. Statistics-based outlier detection for wireless sensor networks. Int J Geogr Inf Sci. 2012;26(8):1373–92.

14. Hill D, Minsker B. Anomaly detection in streaming environmental sensor data: a data-driven modeling approach. Environ Model Softw. 2010;25(9):1014–22.

15. Munir M, Siddiqui S, Dengel A, Ahmed S. DeepAnT: a deep learning approach for unsupervised anomaly detection in time series. IEEE Access. 2019;7:1991–2005.

16. Zhou Y, Arghandeh R, Spanos C. Online learning of contextual hidden Markov models for temporal-spatial data analysis. 2016 IEEE 55th Conf Decis Control CDC 2016. 2016;6335–41.

17. Sakurada M, Yairi T. Anomaly detection using autoencoders with nonlinear dimensionality reduction. ACM Int Conf Proceeding Ser. 2014;02:4–11.

18. Cheng H, Tan P, Potter C, Klooster S. Detection and characterization of anomalies in multivariate time series. Proceedings. 2009;1:409–20.

19. Cheng H, Tan P, Potter C, Klooster S. A robust graph-based algorithm for detection and characterization of anomalies in noisy multivariate time series. Proc - IEEE Int Conf Data Min Work ICDM Work 2008. 2008;349–58.
